# Co-inhibition of pol θ and HR genes efficiently synergize with cisplatin to suppress cisplatin-resistant lung cancer cells survival

**DOI:** 10.18632/oncotarget.11214

**Published:** 2016-08-11

**Authors:** Chun-Hua Dai, Ping Chen, Jian Li, Tin Lan, Yong-Chang Chen, Hai Qian, Kang Chen, Mei-Yu Li

**Affiliations:** ^1^ Department of Radiation Oncology, Affiliated Hospital of Jiangsu University, Zhenjiang, China; ^2^ Department of Pulmonary Medicine, Affiliated Hospital of Jiangsu University, Zhenjiang, China; ^3^ Institute of Medical Science, Jiangsu University, Zhenjiang, China

**Keywords:** lung cancer cells, cisplatin-resistance, translesion synthesis, pol θ, homologous recombination

## Abstract

Cisplatin exert its anticancer effect by creating intrastrand and interstrand DNA cross-links which block DNA replication and is a major drug used to treat lung cancer. However, the main obstacle of the efficacy of treatment is drug resistance. Here, we show that expression of translesion synthesis (TLS) polymerase Q (POLQ) was significantly elevated by exposure of lung cancer cells A549/DR (a cisplatin-resistant A549 cell line) to cisplatin. POLQ expression correlated inversely with homologous recombination (HR) activity. Co-depletion of BRCA2 and POLQ by siRNA markedly increased sensitivity of A549/DR cells to cisplatin, which was accompanied with impairment of double strand breaks (DSBs) repair reflected by prominent cell cycle checkpoint response, increased chromosomal aberrations and persistent colocalization of p-ATM and 53BP1 foci induced by cisplatin. Thus, co-knockdown of POLQ and HR can efficiently synergize with cisplatin to inhibit A549/DR cell survival by inhibiting DNA DSBs repair. Similar results were observed in A549/DR cells co-depleted of BRCA2 and POLQ following BMN673 (a PARP inhibitor) treatment. Importantly, the sensitization effects to cisplatin and BMN673 in A549/DR cells by co-depleting BRCA2 and POLQ was stronger than those by co-depleting BRCA2 and other TLS factors including POLH, REV3, or REV1. Our results indicate that there is a synthetic lethal relationship between pol θ-mediated DNA repair and HR pathways. Pol θ may be considered as a novel target for lung cancer therapy.

## INTRODUCTION

Platinum-based chemotherapy agents, such as cisplatin, are first-line treatment drugs of advanced non-small cell lung cancer (NSCLC). The anticancer effect of cisplatin is though its ability to covalently interact with guanine residues in DNA resulting in the formation of both intrastrand and interstrand DNA cross-links (ICLs) [1, 2]. Although ICLs comprised only a small fraction of the induced DNA damage, these are the most cytotoxic and genotoxic lesions produced by cisplatin [2–4]. However, the effectiveness of the therapy is often compromised largely because cancer cells develop resistance to the drug [3, 4]. Multiple mechanisms that mediate intrinsic or acquired resistance to cisplatin have been identified, including decreased drug uptake, increase of drug metabolism and inactivation, defects in apoptosis programs, and enhanced DNA repair capacity [5, 6]. Enhanced DNA repair pathways are found in a subset of drug-resistant cancer cells [7–10]. Therefore, DNA damage repair is one of main cisplatin resistant mechanisms.

Repair of ICLs requires the coordination of multiple DNA repair pathways including Fanconi anemia (FA), Homologous recombination (HR), translesion synthesis (TLS) pathways, and endonuclease-mediated DNA processing [11–14]. The FA pathway is composed of at least 20 genes, which are named FANCA through FANCU. The proteins encoded by these genes act cooperatively in the FA pathway to coordinate the repair of DNA ICLs [11]. The eight upstream FA factors and several FA associated proteins (including FAAP20) assemble into FA core complex which monoubiquitinate FANCD2 [12]. Monoubiquitinated FANCD2 recruits some endonuclease to the site of DNA damage to make incisions on different intermediates in ICL repair, which convert replication forks stalled at ICLs to double strand breaks (DSBs) to initiate HR-mediated repair [15–17]. Importantly, several HR components are part of the FA pathway. For instance, FANCD1/BRCA2 and FANCO/RAD51C, which are the FA pathway downstream factors and mainly facilitate the loading of RAD51 to initiate the HR process [18–20]. TLS, carried out by a numerous mutagenic DNA polymerases, such as Pol η (encoded by POLH), Pol ζ (consisting of the catalytic subunit REV3 and the structural subunit REV7), and REV1, protects the genome from large deletion by replicating across ICLs and other occluding lesions [13, 21–23]. TLS polymerases are essential for ICL repair to bypass an ICL unhooked from one of the two cross-links strands [11, 12]. Pol ζ and REV1 are key factors in ICL repair, as cells deficient in either one of these genes are exquisitely sensitive to cross-linking agents [24, 25]. Human cells deficient in Pol η are also hypersensitive to cross-linking agents such as cisplatin and psoralen [26, 27]. However, the role of Pol θ (encoded by POLQ) in the cell is still a matter of debate. In vertebrate cells, there are conflicting reports concerning the sensitivity of Pol θ to ICL-inducing agents. DT40 cells lacking Pol θ were not hypersensitive to cisplatin [28, 29]. But depletion of POLQ can sensitized mouse CH12B-lymphama cells to cisplatin and mitomycin (MMC) [30, 31]. The chaos 1 (a mutant allele of POLQ) mutant mice and cells derived from them exhibited no hypersensitivity to MMC, implying that POLQ does not participate in the repair of ICLs *in vivo* [32]. Accumulating evidence suggests a role for POLQ in the repair or tolerance of DSBs. Mouse bone marrow cells deleted for POLQ are more sensitive than normal cells to ionizing radiation (IR) and bleomycin, both of which are known to produce DSBs [33]. Depleting of POLQ in human cancer cells caused an increase in IR-induced γH2AX foci and sensitized the cells to γ-irradiation [34]. Recent studies showed that pol θ participated in microhomology mediated end-joining (MMEJ) which is an error-prone alternative DSB repair pathway that utilizes sequence microhomology to recombine broken DNA [35–38]. Whether Pol θ interacts with classical DNA repair pathways to offer cisplatin resistance remains unknown. In the present study, we examine the contribution of Pol θ to cisplatin resistance in NSCLC cells in comparison with Pol η, REV3 and REV1, and investigate whether Pol θ is involved in repair and tolerance of cisplatin-induced DNA damage in cooperation with HR.

## RESULTS

### POLQ expression was markedly higher upon cisplatin exposure in A549/DR cells

**Figure 1:**
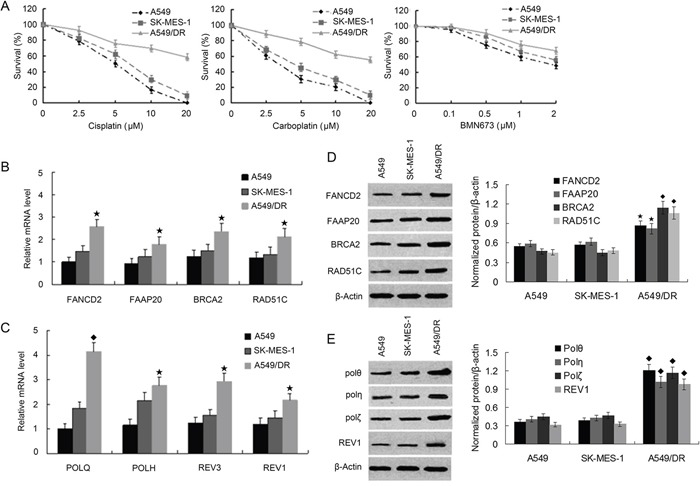
A549/DR cells are resistant to cross-linking agents, and expression of FA, HR and TLS pathway factors are elevated compared with A549 and SK-MES-1 cells **A.** A549, SK-MES-1, and A549/DR cells growing in 96-well plates were treated with cisplatin, carboplatin and BMN673 at the indicated dose. The CCK-8 assay was used to determine cell survival. **B.** and **C.** Total RNA was isolated from A549, SK-MES-1 and A5491DR cells. RNA was subjected to real time quantitative-PCR to determine the mRNA levels of the FA, HR and TLS pathway factors as the indicated. (★ compared with A549 and SK-MES-1 cells, P < 0.05; ◆ compared with A549 and SK-MES-1 cells, P < 0.01). **D.** and **E.** Whole cell lysate was prepared from the A549, SK-MES-1 and A 549/DR cells and subject to Western blot with specific antibodies as the indicated to determine the protein levels of various FA, HR and TLS pathway factors (◆ compared with A549 and SK-MES-1 cells, P < 0.05; ★ compared with A549 and SK-MES-1 cells, P < 0.01).

**Figure 2:**
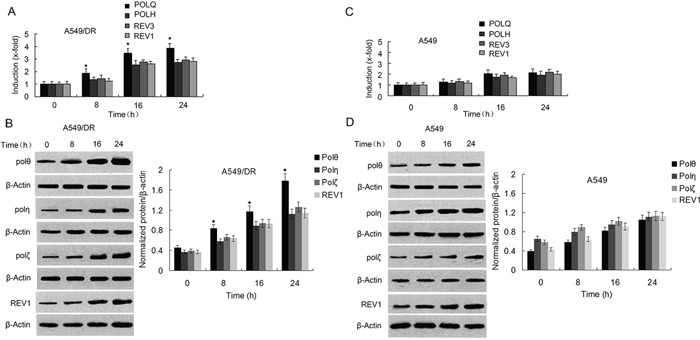
Expressions of POLQ were significantly increased in A549/DR cells compared with POLH, REV3, and REV1 by exposure of the cells to cisplatin **A.** and **C.** Real-time quantitative-PCR was performed to determine mRNA expression of TLS pathway factors as indicated in A549/DR and A549 cells at different time points after cisplatin treatment. The expression of POLQ was normalized to GAPDH; the untreated control was set to one. (★ compared with POLH, REV3 and REV1, P < 0.05). **B.** and **D.** Protein expression of TLS pathway factors as the indicated was analyzed by Western blot using specific antibodies in whole cell lysate of A549/DR and A549 cells after cisplatin treatment. β-actin was used as loading control (◆ compared with Pol η, Pol ζ and REV1, P < 0.01).

To determine whether enhanced DNA crosslink repair in lung cancer may underlie the mechanism of cisplatin-resistance, we chose to use the cisplatin-resistant NSCLC cell line A549/DR which were generated by continuous exposure of A549 cells to increasing concentration of cisplatin for a 10 month period. We compared the cell survival of A549/DR cells with A549 and SK-MES-1 cells (a lung squamous cell carcinoma line) after treatment with cisplatin, carboplatin, or BMN673 (a PARP inhibitor). As expected, A549 cells survival was significantly decreased than that of A549/DR cells following treatment with cisplatin or carboplatin (Figure 1A). A549 cells were only slightly more sensitive to BMN673 than A549/DR cells. In addition, SK-MES-1 cells were more sensitive to cisplatin than A549/DR cells. Similar results were observed in colony formation assay when the three cell lines were treated with same drugs (Supplementary Figure S1A). To determine the role of POLQ in A549/DR cell resistance to cisplatin, we detected the mRNA and protein expression of POLQ and FA, HR, and other TLS factors including FANCD2, FAAP20, BRCA2, RAD51C, POLH, REV3, and REV1. The results showed that the mRNA and protein expressions of these TLS and HR factors in A549/DR cells were elevated as compared with A549 and SK-MES-1 cells (Figure 1B to 1E). However, elevated extent of POLQ expression was more significant than that of FA, HR and other TLS factors in A549/DR cells. To investigate molecular mechanism underlying the protective effect of Pol θ on A549/DR cells upon treatment with cisplatin, the time-dependent expressions of POLQ mRNA was examined by real-time quantitative (RTQ)-PCR. Increased expression of POLQ mRNA was detectable 8 hours after cisplatin treatment and was constantly increasing during the 24-hour post-incubation period (Figure 2A). Induction of POLQ mRNA was accompanied by an increase in the levels of Pol θ protein (Figure 2B). Meantime, time-dependent elevations of POLH, REV3, or REV1 in both mRNA and protein levels were observed in A549/DR cells following cisplatin treatment, but the raised extent of these TLS factor expressions was markedly lower than that of POLQ (Figure 2A and 2B). Additionally, increases of these TLS factor expression were not obvious in A549 and SK-MES-1 cells after cisplatin treatment (Figure 2C and 2D, and Supplementary Figure S1B-S1D). The findings suggest that Pol θ may play a more important role in acquired resistance of A549/DR cells to cisplatin.

### Impact of Pol θ on the sensitivity to cisplatin in A549/DR cells

**Figure 3:**
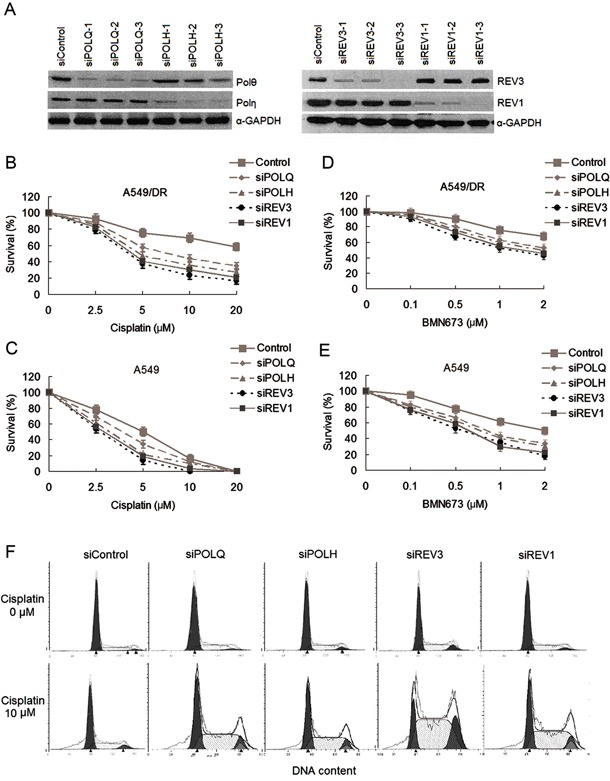
The changes of sensitivity to cisplatin and BMN673 in A549/DR cells and A549 cells after transfections of siRNAs against to TLS pathway factors **A.** Validation of siRNAs used in this study. Representative western blot showing POLQ, POLH, REV3 and REV1 expression in A549/DR and A549 cells. Cells were transfected with the indicated siRNAs for 48 hours. Whole cell lysates were prepared and subjected to Western blot for detecting the protein expressions of these factors. **B.** and **D.** A549/DR cell and **C.** and **E.** A549 cells growing in 96-well plates were transfected with various siRNA as indicated. Cell survival was determined by CCK-8 assay following cisplatin or BMN673 treatment. **F.** A549/DR cells depleted of POLQ, POLH, REV3 or REV1 exhibit a cisplatin-induced cell cycle arrest in S/G2 phases. The cells were exposure to 10 μm cisplatin and subject to cell cycle analysis 24h later by flow cytometry.

Since expression levels of POLQ mRNA and protein are higher than those of POLH, REV3 and REV1 in A549/DR cells following exposure to cisplatin, we expected that depletion of POLQ in the cells would result in more hypersensitivity to cisplatin compared with knockdown of other TLS factors by siRNA transfections (Figure 3A). By contrast, we found that POLH, REV3, or REV1 siRNA-transfected A549/DR cells exhibited greater sensitivity to cisplatin-induced cytotoxicity, although transfection of siRNA against POLQ also sensitize A549/DR cells to cisplatin (Figure 3B). Similarly, A549 cells depleting POLH, REV3 or REV1 were more sensitive to cisplatin compared to the cells depleted of POLQ (Figure 3C). Depleting SK-MES-1 cells the four TLS factors achieved the same results as A549 cells (Supplementary Figure S1E). In addition, we examined the impact of TLS factors on sensitivity to PARP inhibitor in the lung cancer cells, and fond that knockdown of the four TLS factors slightly enhanced sensitivity of A549/DR and A549 cells to BMN (Figure 3D and 3E). Meanwhile, A549/DR cells depleted of POLQ, POLH, REV3 or REV1 after cisplatin treatment generated cell cycle arrest in S and G2 phases compared to the cells without cisplatin treatment (Figure 3F).

**Figure 4:**
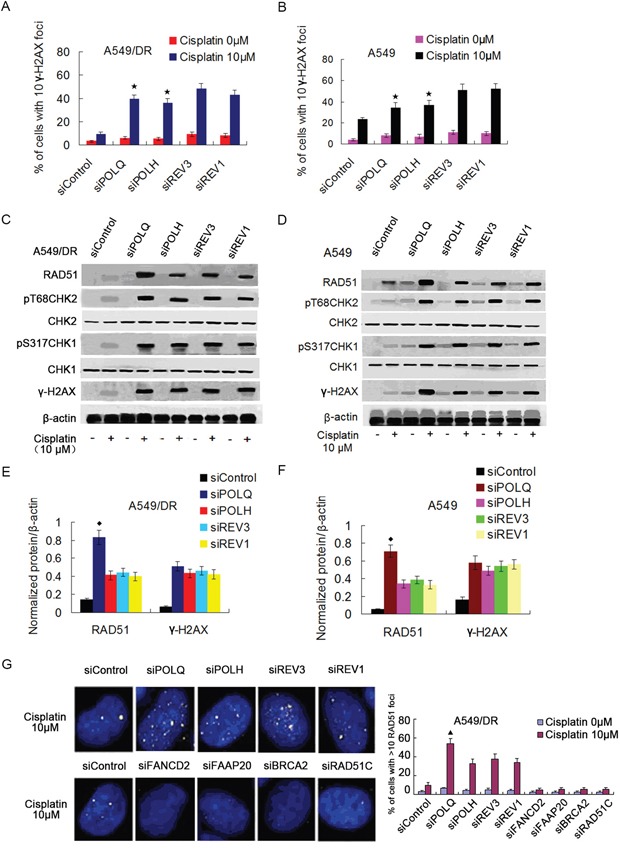
A549/DR cells depleted of POLQ, POLH, REV3 or REV1 display significant DNA damage response, and depletion of POLQ remarkably enhance RAD51 expression **A.** and **B.** A549/DR and A549 cells were treated with indicated dose of cisplatin, and fixed and immunostained with γH2AX antibody. The percentage of cells with > 10 γH2AX foci was shown as the mean ± SEM from three independent experiments (★ compared with siREV3 and siREV1, P < 0.05). Additional representative images are shown in Supplementary Figure S2. **C-F.** siRNA transfected A549/DR and A549 cells were treated with cisplatin at indicated dose for 2 hours, cells were harvested and subject to Western blot with antibodies as indicated (◆ compared with siPOLH, siREV3 and siREV1, P < 0.005). **G.** siRNA transfected A549/DR cells were treated with indicated dose of cisplatin, and fixed and immunostained with RAD51 antibody. The percentage of cells with >10 RAD51 foci was quantified from Image Software (▲ compared with siPOLH, siREV3 and siREV1, P < 0.01).

Activation of ATM and ATR kinases are well characterized response to DNA damage such as DSBs or replication fork stalling [39, 40]. Therefore, we measured the phosphorylation of ATM and ATR substrates (e.g., H2AX, CHK1 and CHK2) as surrogate markers for DSBs and replication stress as a result of deficient TLS [41, 42], and examined whether depletion of POLQ, POLH, REV3 or REV1 in A549/DR and A549 cells lead to replication stalling and activations of ATM and ATR after cisplatin treatment. The results showed that knockdown of POLQ, POLH, REV3 or REV1 in the two cell lines strikingly increased the intensity of γH2AX in term of expression levels and the percentage of cells with 10 γH2AX foci following cisplatin treatment (Figure 4A to 4F, and Supplementary Figure S2A and S2B). In line with the results of cell survival analysis, the increase of γH2AX foci formation in cells depleting REV3 or REV1 was more obvious compared with cells lacking POLQ or POLH (Figure 4A and 4B). Enhanced phosphorylations of CHK1 and CHK2 cell cycle checkpoint kinases were found in the two cell lines depleted of POLQ, POLH, REV3 or REV1, but levels of phosphorylated CHK1 and CHK2 did not differ between cells depleted of POLQ or POLH and REV3 or REV1 knockdown cells (Figure 4C to 4F). These finding imply that POLQ may have additional mechanism in promoting tolerance and resistance to cisplatin in addition to bypassing DNA adduct.

### POLQ expression correlated inversely with HR activity in A549/DR cells

**Figure 5:**
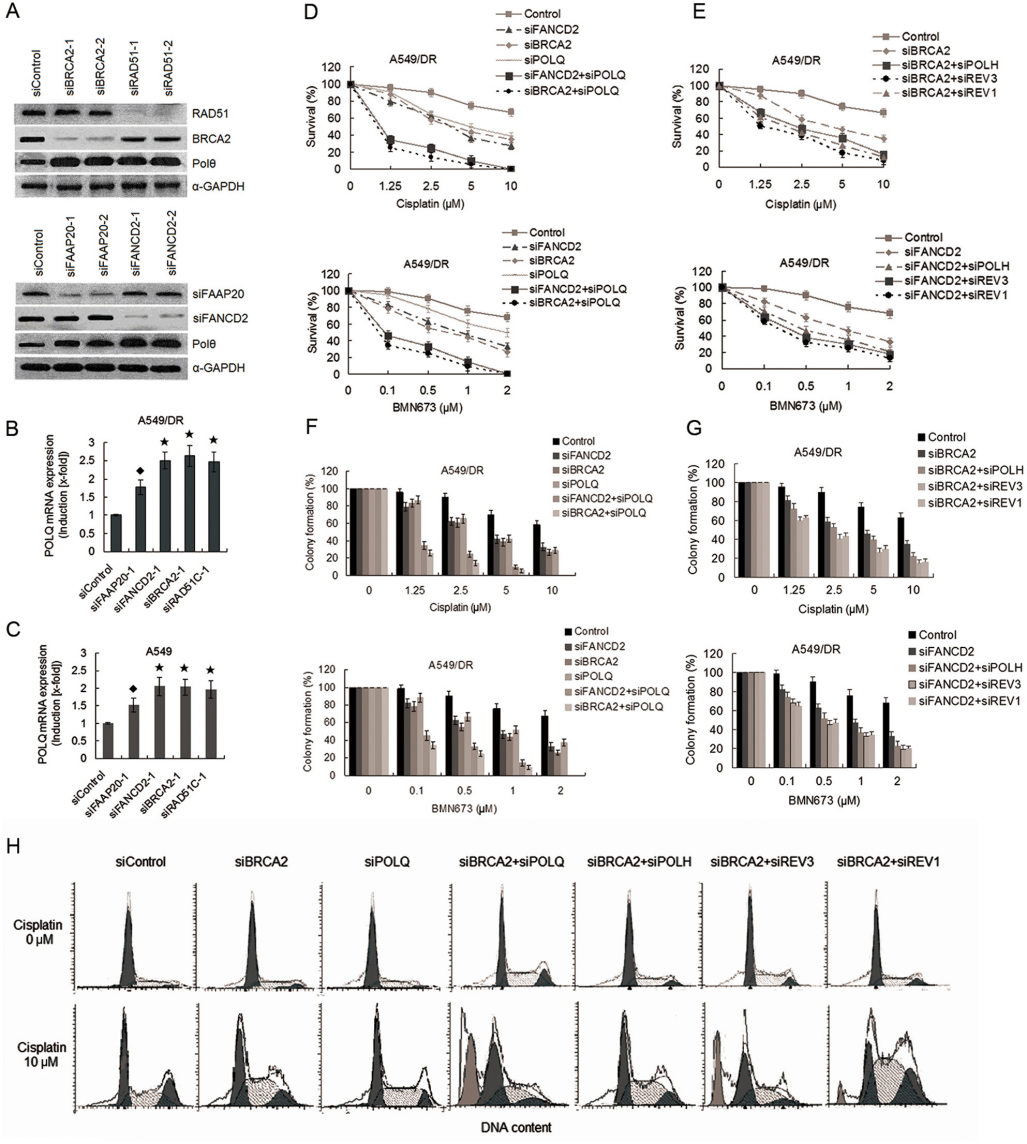
Co-depletion of POLQ and FANCD2 or BRCA2 markedly increase sensitivity of A549/DR cells to cisplatin and BMN673 compared with double depletion of BRCA2 and POLH, or REV3, or REV1 **A.** Representative western blot showing BRCA2, RAD51, FAAP20 and FANCD2 expression in A549/DR cells after siRNA transfections. Expressions of Pol θ were markedly increased after transfection with siRNAs against FANCD2, FAAP20, BRCA2, and RAD51C. **B.** and **C.** Expressions of POLQ mRNA in A549/DR and A549 cells were significantly elevated after transfection with siRNAs against FANCD2, FAAP20, BRCA2, and RAD51C. Real-time quantitative-PCR was used to determine mRNA expressions. (★ compared with siControl, P < 0.001; ◆ compared with siControl, P < 0.01). **D.** and **E.** A549/DR cells were treated with cisplatin or BMN673 at the indicated dose following transfection with various siRNAs as indicated. Then cell survival was determined by the CCK-8 assay. **F.** and **G.** A549/DR cells were treated with cisplatin or BMN673 at the indicated dose following transfection with various siRNAs as indicated. The cells were then stained by crystal violet and total colonies were counted after two weeks. Colony numbers of control-treated cells were set as 100%. **H.** Co-depletion of BRCA2 and POLQ result in dramatically increased sub-G1 cells in response to cisplatin. A549/DR cells transfected with siRNAs as indicated were exposure to cisplatin, and subject to cell cycle analysis by flow cytometry.

Previous studies indicated that POLQ was implicated in the tolerance or repair of DSBs induced by cisplatin. We then assess the relationship between POLQ expression and HR. We found that knockdown of POLQ in A549/DR and A549 cells caused a remarkably increase of RAD51 in term of expression levels and number of cells with RAD51 foci (Figure 4C to 4G, and Supplementary Figure S2C and S2D). Although POLH, REV3 or REV1-depleted A549/DR and A549 cells also displayed higher RAD51 expression levels and more numbers of cells with RAD51 foci than siControl cells, which is similar to POLH, REV3, or REV1 knockdown HeLa cell displaying raised RAD51 foci number after exposure to ionizing radiation (IR) [43], the increase of expression levels and foci formation of RAD51 were inferior to POLQ-depleted A549/DR and A549 cells (Figure 4C to 4G and Supplementary Figure S2C and S2D). On the other hands, siRNA-mediated inhibition of HR genes including BRCA2, RAD51C, FAAP20 or FANCD2 increased POLQ expression in mRNA and protein levels (Figure 5A to 5C), taken together, suggesting that POLQ expression correlated inversely with HR activity, and lung cancer cells with higher-POLQ expression may be companied with HR deficiency, which is agree with the findings in epithelial ovarian cancer cells reported by Cacceldi et al [44].

### Co-depletion of POLQ and HR genes efficiently synergize with cisplatin to suppress A549/DR cells survival

To determine whether there is the synthetic lethality between POLQ and HR genes, we performed cell survival assay in A549/DR and A549 cells treated with cisplatin or BMN673 (a PARP inhibitor) following co-transfection with siRNAs targeting POLQ and HR genes. The transfection efficiency was verified in parallel experiments by Western blot analysis (Figure 3A and Figure 5A). The results shown that co-knockdown of POLQ and BRCA2, or FANCD2 in the two cell lines resulted in hypersensitivity to cisplatin as compared with individual depletion of FANCD2, BRCA2, or POLQ (Figure 5D and Supplementary Table S1A). Similar results were found in colony formation assay (Figure 5F). Moreover, the 50% inhibitory concentrations (IC50) of cisplatin in A549/DR co-depleting POLQ and BRCA2, or FANCD2 were even lower than those in A549 cells with the same gene depletions, indicating that the sensitization effect of co-knockdown of POLQ and BRCA2, or FANCD2 in A549/DR cells was stronger than in A549 cells (Supplementary Figure S3B and Supplementary Table S1A). Similarly, A549/DR cells co-depleted of POLQ and FANCD2 or BRCA2 were more sensitive to BMN673 than those depleting FANCD2, or BRCA2, or POLQ alone (Figure 5D and Supplementary Table S1B). Also, the sensitization to BMN673 in A549/DR cells by co-depleting POLQ and BRCA2 or FANCD2 was more significant than those in A549 cells (Supplementary Figure S3B and Supplementary Table S1B).

We further assess the impact of co-knockdown of HR and other three TLS genes on cisplatin-induced cytotoxicity. The results showed that the A549/DR cells co-depleted of both BRCA2 and POLH, or REV3, or REV1 were more sensitive to cisplatin or BMN673 than the cells depleting BRCA2 alone (Figure 5E, and Supplementary Table S1C and S1D). Importantly, suppression of survival in A549/DR cells co-depleted of BRCA2 and POLQ were more significant than in the cells co-depleted of both BRCA2 and POLH, or REV3, or REV1 after treatment with cisplatin or BMN673 (Figure 5D and 5E, and Supplementary Table S1C and S1D). A549 cells co-depleted of BRCA2 and POLQ did not show the sensitization effect like A549/DR cells to cisplatin and BNM673 (Supplementary Figure S3C and Supplementary Table S1C and S1D). Meanwhile, cell cycle analysis showed that double knockdown of BRCA2 and POLQ, or POLH, or REV3, or REV1 in A549/DR cells evoked prominent cisplatin-induced S/G2 arrest, but the cells co-depleted of BRCA2 and POLQ exhibited notably increased levels of death as reflected by emerging more Sub-G1 cells in response to cisplatin (Figure 5H).

### Impact of co-depletion of POLQ and HR genes on repair of cisplatin-induced DNA damage

**Figure 6:**
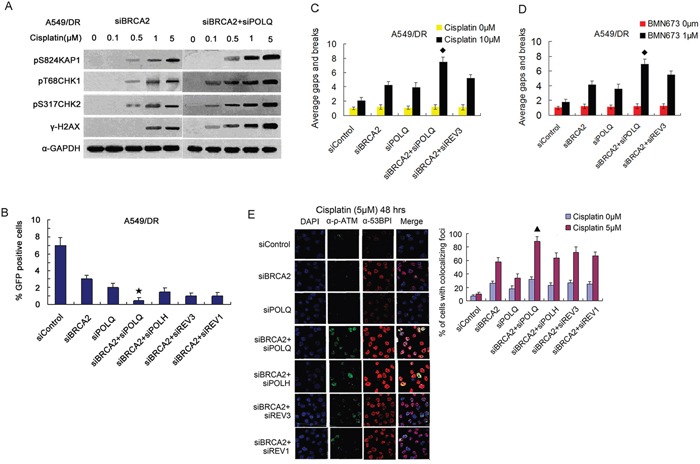
Co-depletion of BRCA2 and POLQ in A549/DR cells caused strikingly cisplatin-induced cell cycle checkpoint response, and an inhibition of HR, and increased cisplatin-induced P-ATM and 53BP1-colocalized foci **A.** A549/DR cells co-depleted of BRCA2 and POLQ display notably enhanced cisplatin-induced phosphorylation of H2AX, CHK1, CHK2 and KAP1 proteins, and **B.** show a significant decrease of percentage of GFP positive cells (★ compared with siBRCA2, siPOLQ, siBRCA2 +siPOLH, siBRCA2+siREV3 and siBRCA2+siREV1, P < 0.05), and **C.** a marked increase of cisplatin-induced, and **D.** BMN673-induced chromosomal aberrations (◆ compared with siBRCA2, siPOLQ and siBRCA2+siREV3, P < 0.05), and **E.** exhibit markedly increased percentage of cells with P-ATM and 53BP1-colocalized foci after cisplatin treatment (▲ compared with siBRCA2, siPOLQ, siBRCA2+siPOLH, siBRCA2+siREV3, and siBRCA2+siREV3, P < 0.05).

Since POLQ and HR factors are involved in the repair of DSBs, and POLQ expression correlated inversely with HR activity, we investigated whether POLQ cooperate with HR genes in repairing DNA damage produced by cisplatin. Western blot assay showed that co-depletion of BRCA2 and POLQ caused dramatically potentiated phosphorylation of H2AX, CHK1 and CHK2 compared with BRCA2 depletion alone in A549/DR and A549 cells after cisplatin treatment (Figure 6A and Supplementary Figure S3D). Similar results were observed when phosphorylation of KAP1 on Ser-428 by ATM and ATR kinases, another marker for DNA damage response [45], was analyzed (Figure 6A and Supplementary Figure S3D). Furthermore, co-depletion of BRCA2 and one of the four TLS factors markedly inhibited HR repair of I-SceI induced DSB as indicated by GFP positive cells (Figure 6B and Supplementary Figure S4A). But the most significant reduction in gene conversion frequency was observed in A549/DR cells co-depleted of BRCA2 and POLQ (Figure 6B and Supplementary Figure S4A). Consistent with an inability to complete HR repair of cisplatin-induced DSBs, BRCA2 and POLQ co-depleted A549/DR cells displayed a dramatic increase in cisplatin-induced chromatid gaps and breaks per metaphase compared to the cells depleting BRCA2 or POLQ alone and the cells co-depleting BRCA2 and REV3 (Figure 6C and Supplementary Figure S4B).

Localization of activated ATM protein kinase and 53BP1 to DSB are both well characterized surrogate markers of DSBs [41, 46]. Therefore, we test the formation of foci marked by activated ATM colocalized with 53BP1 in cisplatin-treated A549/DR cells. The results showed that the percentage of BRCA2 and POLQ co-depleted cells exhibiting P-ATM and 53BP1-colocalized foci persisted at higher levels 48 hours after cisplatin treatment, suggesting that DSB repair in these cells was affected to a larger degree, compared to the cells depleting BRCA2 or POLQ alone, and the cells co-depleted of BRCA2 and POLH, or REV3, or REV1 (Figure 6E). In addition, co-depletion of BRCA2 and POLQ also led to a significant elevation of chromatid gaps and breaks per metaphase in BMN673-treated A549/DR cells (Figure 6D and Supplementary Figure S4B). In line with a prominent increase of chromosome aberration, co-depletion of BRCA2 and POLQ resulted in notably enhanced γ-H2AX staining by immunofluorescence post-treatment with BMN673 (Supplementary Figure S4C).

## DISCUSSION

An increasing amount of evidence indicate that DNA repair ability is one of main determinants in offering chemoresistance to cisplatin, and the development of cisplatin resistance is a dynamic process involving multiple DNA repair pathway [5, 6]. Here, we show that A549/DR cells, a cisplatin-resistant lung cancer cell line, exhibited increased expression levels of FA, HR and TLS pathway factors compared with their parent cell line A549 and another lung cancer cell line SK-MES-1 which is relative sensitivity to cisplatin. However, the increased extent of POLQ in both mRNA and protein levels in A549/DR cells were more obvious than other TLS factors including POLH, REV3 and REV1. Furthermore, induction of POLQ expression by cisplatin in A549/DR cells reached the highest levels among the TLS factors tested in this study, suggesting that POLQ may play a more important role in generation of acquired cisplatin resistance in A549/DR cell. However, the results of cell survival assay did not support this conjecture, in which the sensitization effect to cisplatin in A549/DR cells by depleting POLQ was inferior to that in the cells deficient in POLH, or REV3, or REV1. The percentage of γH2AX foci positive A549/DR cells depleting POLQ was lower than the cells depleted of REV3 or REV1, although cells individually depleted of POLQ, POLH, REV3, or REV1 displayed similar and enhanced cell cycle checkpoint response, as measured by the phosphorylated H2AX, CHK1 and CHK2 kinase expression.

It is now recognized that the cooperation action of Pol η Pol ζ and REV1 is required for replicative bypass of DNA intrastrand cross-links, including those generated by cisplatin [26, 47–49]. What is more, Polζ and REV1 are necessary for the repair of cisplatin interstrand cross-links and DSBs caused by cisplatin, MMC or IR [42, 43]. In fact, Pol θ is also very efficient at incorporating nucleotides opposite abasic sites and then extending past the lesion [50, 51], and is involved in the repair of DSBs produced by cisplatin, etoposide, bleomycin, and IR [30, 33, 34]. In this study, further analysis show that depletion of POLQ in A549/DR and A549 cells remarkably increased RAD51 expression and its foci formation, and the inhibition of HR pathway by depleting BRCA2 or RAD51C increased Pol θ expression, which is accordance with the results reported by Ceccaldi et al [44]. These findings suggest that Pol θ in lung cancer cells suppress HR activity and participate in DSB repair through alternative pathway. We showed that co-depletion of POLQ and BRCA2 or FANCD2 significantly increased sensitivity of A549/DR cells to cisplatin compare to individual depletion of BRCA2, or FANCD2, or POLQ. Moreover, the sensitization effects to cisplatin by co-depleting BRCA2 and POLQ in A549/DR cells were stronger than those in the cells co-depleted of both BRCA2 and POLH, or REV3 or REV1, indicating that there is a synthetic lethal relationship between Pol θ-mediated DNA repair and HR pathway in A549/DR cells. In addition, inhibition of survival induced by cisplatin in A549/DR cells co-depleted of BRCA2 and POLQ was greater than that in A549 cells co-depleted of BRCA2 and POLQ, further supporting this notion. Consistent with the notion is the findings that the hypersensitization effect to cisplatin by co-depleting POLQ and BRCA2 in the cells was associated with a potentiated cell cycle checkpoint response and a marked increase in cisplatin-induced chromosome aberrations. And co-depleting A549/DR cells of BRCA2 and POLQ led to a greater decrease in HR repair produced by the I-SceI, reflected by measuring GFP positive cells, compared to individual knockdown of BRCA2 or POLQ, and double knockdown of BRCA2 and POLH, or REV3, or REV1.

It is well known that DSBs are the most lethal lesions induced by cisplatin, and repaired by two major pathways within the cell: HR and non-homologous end-joining (NHEJ) [52]. In addition to these two well-established repair modes, an alternative end-joining pathway (called MMEJ) was recently described [53, 54]. MMEJ promotes inter-and intra-chromosome re-arrangements associated with DNA deletions by utilizing sequence microhomology to recombine broken DNA end [35–39, 53, 54]. Pol θ was recently identified to play a major role in MMEJ of DSBs in C. elegans, mice and human cells [36–38, 44]. MMEJ is generally not the preferred method of DSB repair in health cells, but it is increasingly important in cell deficient in HR [55]. Although the physiologically relevant contexts for when MMEJ is the repair route of choice remains unknown, several studies suggested that Pol θ is important in repairing replication-associated DSBs in cells that fail to bypass endogenous DNA lesions [37] or unwind thermodynamically stable DNA structure [35]. The recent findings that HR-deficient cancer cell are dependent on repair executed by Pol θ suggest that HR and MMEJ can act on similar substrate [44], and there may be a constraint or a complementary relationship between Pol θ and HR pathway, perhaps these studies may explain our findings that co-knockdown of BRCA2 and POLQ can efficiently synergize with cisplatin to inhibit survival of cisplatin-resistant lung cancer cells. Further investigations are required to clarify the mechanisms that there is a synthetic lethal relationship between POLQ-mediated DNA repair and HR pathway.

In this study, another interesting finding is that co-knockdown of POLQ and BRCA2, or FANCD2 caused more notable sensitization effect on BMN673 compared with individual knockdown of BRCA2, FANCD2, or POLQ in A549/DR cells. Corresponding to the result is that the percentage of γH2AX foci positive cells and numbers of chromatid aberrations per metaphase were dramatically elevated in A549/DR cell co-depleted of BRCA2 and POLQ following BMN673 treatment. PARP1 is a protein involved in single-strand break (SSD) repair through base excision repair (BER), and is another key factor in alternative end-joining pathway [56–58]. PARP inhibitors (PARPi) mainly suppress BER, which can result in DSBs and replication fork collapse. Those DSBs can be effectively repaired via the HR pathway. Inhibition of the BER pathway, taken together with deficiency of HR, creates a synthetic lethality, which can be exacerbated when used in conjunction with suppression of alternative end-joining pathway or chemotherapy agents [59, 60]. Therefore our results may be interpreted by the notion that the combination of HR deficiency and Pol θ loss by siRNA transfection with suppression of PARP by PARPi can lead to a more potent effect of synthetic lethality.

In conclusion, we show for the first time that POLQ expression was markedly up-regulated by exposure of cisplatin-resistant NSCLC A549/DR cells to cisplatin. POLQ expression and HR activity were inversely related. Co-depletion of POLQ and HR factors such as BRCA in A549/DR cell resulted in a significant sensitization effect to cisplatin or BMN673, and conduced prominent activation of cell checkpoint kinases and an increase in cisplatin and BMN673-induced chromosomal aberrations. Thus our study identifies novel synthetic lethal interactions between POLQ-mediated DNA repair and HR pathway that may be utilized for NSCLC adjuvant therapy with cisplatin. Before POLQ can be considered as a novel target in NSCLC therapy, it roles in the mechanisms of cisplatin-resistance will need to be further investigated.

## MATERIALS AND METHODS

### Cell lines and materials

A549 (a human lung adenocarcinoma cell line), A549/DR (a cisplatin-resistant A549 cell line), and SK-MES-1 (a human lung squamous cell carcinoma cell line) were purchased from the Shanghai Institute for Biological Science (China). The cells were cultured in RPMT-1640 supplemented with 5% fetal bovine serum, 1% glutamine, 100μl/ml penicillin, 100μl/ml streptomycin. A549/DR cells were routinely maintained in culture media containing 0.5μg/ml cisplatin and growth in drug free media for 3 days before the experiment. Antibodies to the following antigen used in this study include: FANCD2, FAAP20, RAD51c, RAD51, Pol θ, Pol η, REV3, REV1, 53BP1, and gapdh were from Santa Cruz, BRCA2, p-ATM, p-CHK1 (S317), p-CHK2 (T68), p-KAP1(S824) were from Calibiochem, and γH2AX and H2AX were from Cell Signaling. Cisplatin was from Yangtze River Pharmaceutica, Carboplatin were from Qilu Pharmaceutical CO., Ltd, BMN673 was from Selleck Chemicals.

### Real-time quantitative PCR

Total RNA was extracted from various cell specimens using Trizol reagent (Invitrogen). Reverse transcription were conducted using Applied Biosystem's Power SYBR Green PCR Master Mix and the actions were run on an ABI 7500 Fast Real-time PCR system, as previously described [61]. The specific primer sequences of the genes detected in this study are showed in Supplementary Table S2.

### Cell survival measurement and colony formation assay

Cell survival was detected by cell counting-kit (CCK-8) assay according to manufacturer's instruction, as previously described [62]. The IC50 concentration was calculated as the cisplatin or BMN673 concentration that kills 50% of cells of untreated control. For colony formation assay, cells were replaced at a density of 500 cells per well onto a 6-well culture plate in DMEM containing 10% FBS. After two weeks, the cells were fixed with 4% paraformaldehyde for 10 min and then stained by using 0.05% crystal violet in ddH2O for 15 min. Alternatively, for examination of clonogenic ability of the cells with drug treatment, a density of 1000 cells per well seeded onto a 6-well culture plate and the indicated drugs at various dose or vehicle in DMEM containing 10% FBS was added to the cultures at 3 day after seeding. The cultures were continuously maintained for another 7 days and subjected to the colony formation assay. Colonies produced by each cell-group were counted and measured using Image Software.

### Western blot

Cells were exposure to the indicated drugs, and proteins from whole cell lysates were prepared and detected using Western blot assay as previously described [61]. The antibodies used to detect the proteins in this study were described above.

### Transfection with siRNA

Cells were seeded at a density of 10^5^ per well of a 6-well plate. Transfection of siRNA into the cells was carried out with Lipofection 2000 (Invitrogen) according to the manufacturer's protocol, as described previously [61]. The sequences of siRNA targeting FANCD2, FAAP20, BRCA2, RAO51C, POLQ, POLH, REV3 and REV1 are described and characterized with respect to knockdown efficiency (Supplementary Table S3 and Figure 3A and 5A).

### Cell cycle analysis and immunofluorescence

For cell cycle analysis, A549/DR cells were transfected with siRNAs as described above and allowed to recover another 2h. Transfected cells were treated with 5μM cisplatin for 1h and washed, harvested 24h later, and fixed in 70% ethanol. The cells were stained with propidium iodide in the presence of RNase A, and then analyzed on a FACS caliber flow cytometer (Becton Dicknson). For immunofluorescence, siRNA transfected A549/DR cells were treated with cisplatin, and treated with 100% methanol 24h later and stained with anti-γH2AX, anti-RAD51, anti-P-ATM, anti-53BP1 and Alexa Fluor dye-conjugated goat anti-mouse and goat anti-rabbit secondary antibody. Images were taken with an Ax-70 microscope (Olympus) and analyzed using Image-Pro software (Medica Cybernetics). Each experiment was performed in triplicate.

### Analysis of chromosomal aberrations

A549/DR cells were transfected with siRNAs as described above, and treated with cisplatin or BMN763. After 24h, mitotic cells were enriched by the addition of 50ng of Colcemid (Gibco)/ml for 45 min prior to cell harvesting. Cells were treated for 20 min at 37°C with a hypotonic solution consisting of 0.075M KCl and then fixed with 3:1 methanol/acetic acid. Cells were dropped onto slides and allowed to dry for a day, and then the chromosomes were stained with Giemsa prior to analysis. A total of 50 mitotic spreads were analyzed for each treatment. The relative number of gaps and breaks per metaphase was calculated relative to control cells.

### Homologous recombination assay

HR assay was conducted by using a GFP-based method as previously reported [63]. In brief, cells were transfected with various siRNAs as above and treated with 10μM cisplatin for 2 h, and efficiency of HR was assessed by co-transfecting an I-SceI expression plasmid (pCBASce) with a GFP-reporter substrate (DR-GFP). The assay works through gene conversion repair of a DSB caused by I-Sce I digestion, such that the DR-GFP plasmides repair by HR express GFP. Cells were transiently transfected with 1μg of DR-GFP plus 3μg of I-SceI expressing vector or 1μg of DR-GFP plus 3μg of control plasmids (Amaxa Biotechnology). The number of GFP-positive cells was evaluated using the Becton Bicknson FACScan, analyzed with the FlowJo Softeware.

### Statistical analysis

All data were expressed as the mean ± SEM of at least three independent experiments. Data were analyzed for statistical significance by using the 2-tailed unpaired Student *t* tests or Fisher's exact test for categorical data. Values of P < 0.05 were considered significant.

## SUPPLEMENTARY MATERIALS FIGURES AND TABLES


