# Supplementary material for: Co-inhibition of pol θ and HR genes efficiently synergize with cisplatin to suppress cisplatin-resistant lung cancer cells survival
Source: Oncotarget. 2016 Aug 11;7(40):65157–70. doi: 10.18632/oncotarget.11214 (PMC5323145; doi:10.18632/oncotarget.11214)
Supplement: Supplementary file 1 [file oncotarget-07-65157-s001.pdf]

Co-inhibition of pol θ and HR genes efficiently synergize with cisplatin to suppress cisplatin-resistant lung cancer cells survival

SUPPLEMENTARY FIGURES AND TABLES

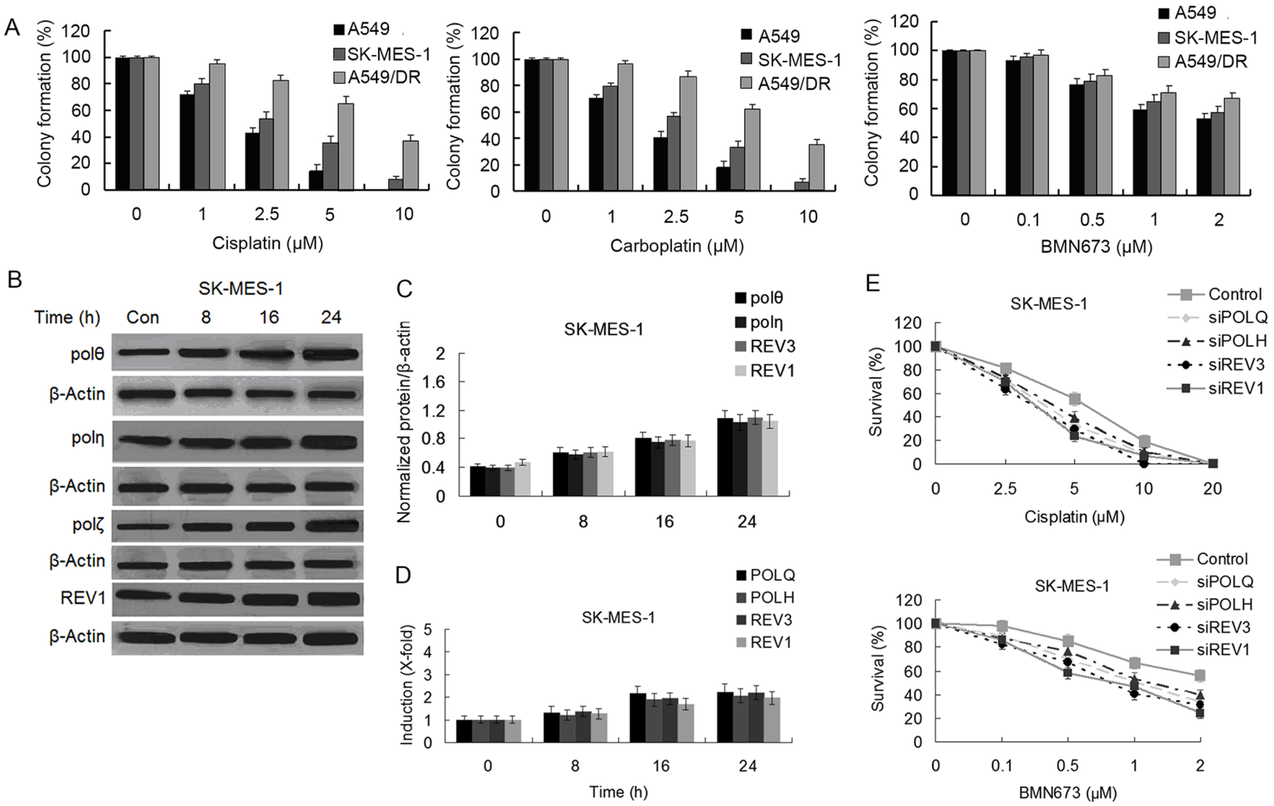

Supplementary Figure S1: A. A549, SK-MES-1, and A549/DR cell were treated with cisplatin, carpoplatin or BMN673, and were then stained by crystal violet. Total colonies were counted after two weeks. Colony numbers of control cells were set as 100%. **B.** and **C.** Western blot was used to determine the protein expression of TLS pathway factors as the indicated in SK-MES-1 cells at different time points after cisplatin treatment. β-actin was used as loading control. **D.** The mRNA expression of TLS pathway factors as indicated was analyzed by real-time quantitative-PCR in SK-MES-1 cells at different time points after cisplatin treatment. The expressions of mRNAs were normalized to GAPDH, the untreated control was set to one. **E.** Cell survival of SK-MES-1 cells was determined by CCK-8 assay following cisplatin or BMN673 treatment. In above experiments, data are present as the mean ± SEM from at least three experiments.

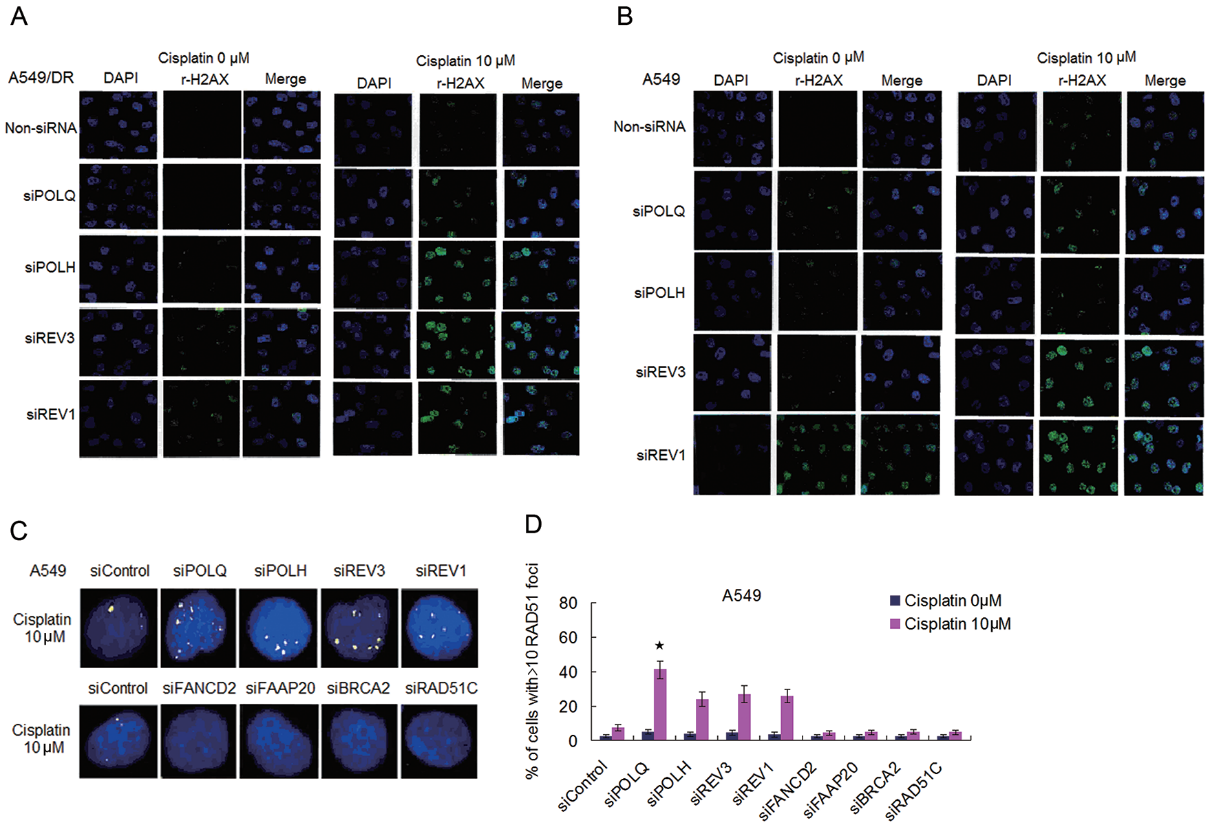

Supplementary Figure S2: A. and **B.** Representative images of γH2AX foci in A549/DR and A549 cells transfected with the indicated siRNAs after treatment with indicated dose of cisplatin. Quantification data are shown in Figure 4. **C.** Representative images of RAD51 foci in A549 cells depleting POLQ, POLH, REV3 and REV1 after treatment with cisplatin. **D.** The percentage of cells with >10 RAD51 foci was quantified from Image Software (★ compared with siPOLH, siREV3, and siREV1, $P < 0.01$).

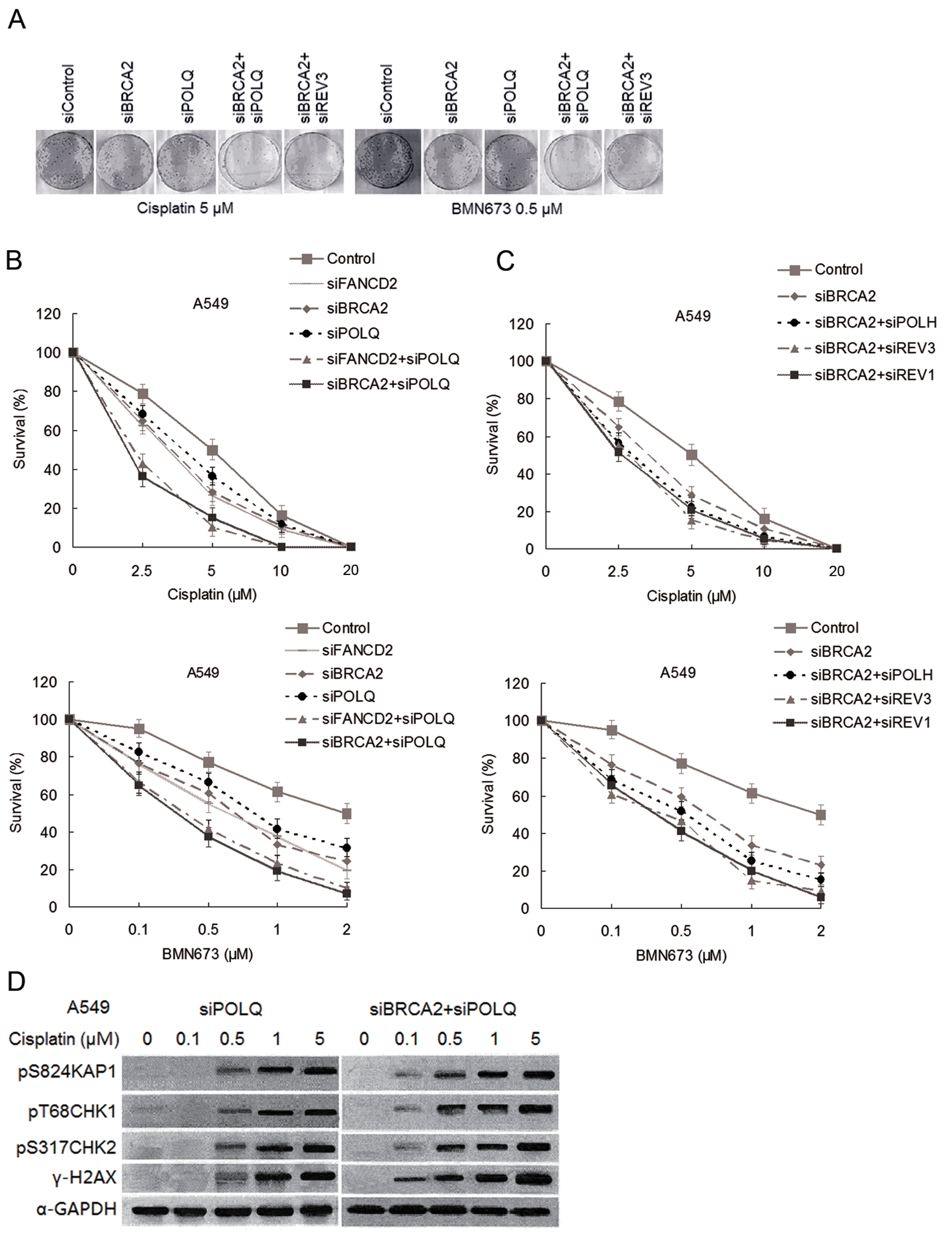

Supplementary Figure S3: A. Representative images of colony formation in A549/DR cells transfected with indicated siRNAs after treatment with cisplatin or BMN673. **B.** and **C.** Cell survival was determined by CCK-8 assay. A549 cells transfected with various siRNAs as indicated were treated with cisplatin or BMN673 at the indicated dose. **D.** A549 cells co-depleting BRCA2 and POLQ display notably enhanced cisplatin-induced phosphorylation of H2AX, CHK1, CHK2 and KAP1 proteins. Western blot was used to detect these phosphorylations.

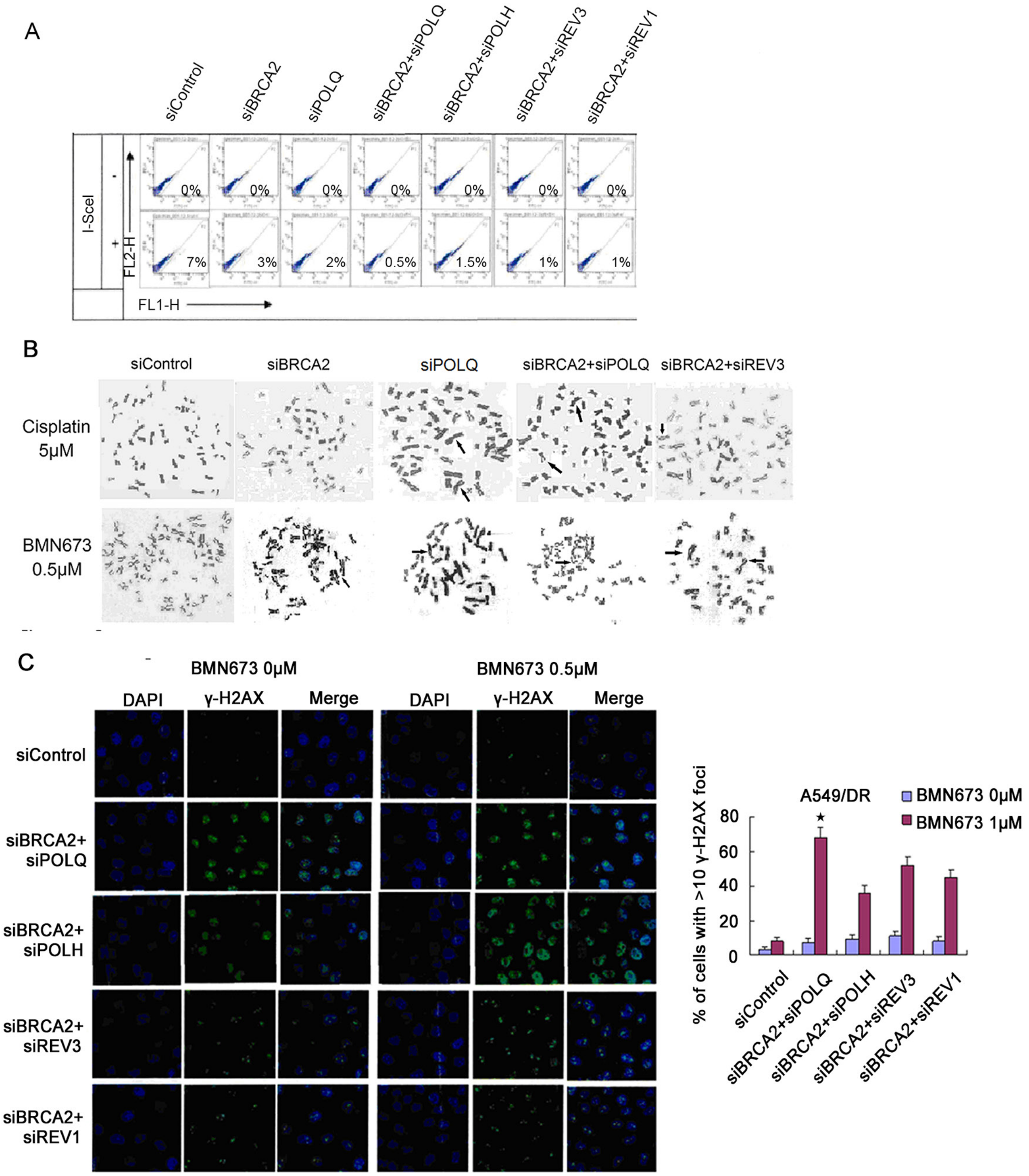

Supplementary Figure S4: A. Representative images of GFP-positive cell expression in A549/DR cells transfected with indicated siRNAs after cisplatin treatment. Quantification data are shown in Figure 6. **B.** Representative images of cisplatin- and BMN673-induced chromatid gaps and breaks in siRNAs transfected A549/DR cells. Quantification data are shown in Figure 6. **C.** Co-depletion of BRCA2 and POLQ in A549/DR cells significantly enhanced BMN673-induced H2AX phosphorylation (★ compared with siBRCA2+siPOLH, siBRCA2+siREV3 and siBRCA2+siREV1, $P < 0.05$).

Supplementary Table S1A: The 50% inhibitory concentration (IC50) of cisplatin in A549/DR and A549 cells

Cell	Control	siFANCD2	siBRCA2	siPOLQ	siFANCD2+ siPOLQ	siBRCA2+ siPOLQ
A549/DR	33.24±3.46	4.05±0.58[a]	4.64±0.62[a]	5.45±0.67[a]	1.12±0.21[a, b]	1.06±0.17[a, b]
A549	5.05±0.44	3.71±0.28[c]	3.74±0.32[c]	3.84±0.35[c]	2.41 ±0.24[c, d]	2.36±0.22[c, d]

[a]Compared with control, p = 0.000
[b]Compared with siFANCD2, or siBRCA2, or siPOLQ, p < 0.005
[c]Compared with control, p < 0.01
[d] Compared with siFANCD2, or siBRCA2, or siPOLQ, p < 0.05

Supplementary Table S1B: The 50% inhibitory concentration (IC50) of BMN673 in A549/DR and A549 cells

Cell	Control	siFANCD2	siBRCA2	siPOLQ	siFANCD2+ siPOLQ	siBRCA2+ siPOLQ
A549/DR	7.54±0.65	1.25±0.14[a]	1.18±0.17[a]	2.45±0.27[a]	0.098±0.018[a, b]	0.093±0.015[a, b]
A549	4.57±0.39	0.65±0.054[c]	0.68±0.062[c]	0.77±0.086[c]	0.30 ±0.035[c, d]	0.27±0.033[c, d]

[a]Compared with control, p = 0.000
[b]Compared with siFANCD2, or siBRCA2, or siPOLQ, p < 0.001
[c]Compared with control, p < 0.001
[d]Compared with siFANCD2, or siBRCA2, or siPOLQ, p < 0.05

Supplementary Table S1C: The 50% inhibitory concentration (IC50) of cisplatin in A549/DR and A549 cells

Cell	Control	siBRCA2	siBRCA2+ siPOLH	siBRCA2+ siREV3	siBRCA2+ REV1	siBRCA2+ siPOLQ
A549/DR	34.05±4.25	4.82±0.53[a]	3.25±0.48[a, b]	2.64±0.36[a, b]	2.67±0.29[a, b]	1.06±0.17[a, c]
A549	4.95±0.54	3.52±0.38[d]	2.66±0.28[d]	2.63±0.25[d]	2.59±0.27[d]	2.36±0.22[d]

[a]Compared with control, p = 0.000
[b]Compared with siBRCA2, p < 0.05
[c]Compared with siBRCA2+siPOLH, siBRCA2+siREV3, siBRCA2+REV1, p < 0.05
[d]Compared with control, p < 0.05

Supplementary Table S1D: The 50% inhibitory concentration (IC50) of BN673 in A549/DR and A549 cells

Cell	Control	siBRCA2	siBRCA2+ siPOLH	siBRCA2+ siREV3	siBRCA2+ REV1	siBRCA2+ siPOLQ
A549/DR	7.35±0.68	1.15±0.12[a]	0.61±0.07[a, b]	0.48±0.065[a, b]	0.52±0.058[a, b]	0.093±0.015[a, c]
A549	4.28±0.45	0.72±0.068[d]	0.58±0.064[d]	0.47±0.054[d]	0.44±0.048[d]	0.27±0.033[d]

[a]Compared with control, p = 0.000
[b]Compared with siBRCA2, p < 0.05
[c]Compared with siBRCA2+siPOLH, siBRCA2+siREV3, siBRCA2+REV1, p < 0.01
[d]Compared with control, p < 0.001

Supplementary Table S2: Primer sequences used for real-time quantitative PCR

Gene	Primer sequences
FANCD2	Forward: ATCTGCTATGATGAATGCTCG<br>Reverse: AGAGCTGCTTTCTTATCACCAAGT
FAAP20	Forwad: GGGCTGCTAATAGGAACACAA<br>Reverse: GCTCGCCGTTATTTTGGATTG
BRCA2	Forward: AATTAGCATGTGAGACCATTGAGA<br>Reverse: GATTTGTGTAACAAGTTGCAGGAC
RAD51C	Forward: ATTGGTACCGCATAAGCATGAAAT<br>Reverse: CGCGCTAGCCGCTGCATTTCAAAG
POLQ	Forward: TATCTGCTGGAACTTTTGCTGA<br>Reverse: CTCACACCATTTCTTTGATGGA
POLH	Forward: AGTCCCGTGGGAAAGCTAACA<br>Reverse: CGAGACATTATCTCCATCACTT
REV3	Forward: CTTTCTCAGATGGCATTCAG<br>Reverse: TTTCGGAACTTGACAGCAGC
REV1	Forward: AAGGCTGATGCAATCGTAA<br>Reverse: CCACCTGGACATTGTCAAGAA

Supplementary Table S3: siRNA target sequences

Gene	siRNA Sequences
siControl	AATTCTCCGAACGTGTCACGT
siPOLQ-1	AGCTTCCACTCCTAGAAGGGA
siPOLQ-2	AAGCCAATTCCATTTAGTTCT
siPOLH-1	CTGGTTGTGAGCATTCGTGTA
siPOLH-2	CAGCCAAATGCCCATTCGCAA
siREV3-1	CGGGATGTAGTCAAACTGCAA
siREV3-2	CCCACTGGAATTAATGCACAA
siREV1-1	AGGAGATATGTCAGTATTGAA
siREV1-2	CAGCGCATCTGTGCCAAAGAA
siFANCD2-1	CAGAGTTTGCTTCACTCTCTA
siFANCD2-2	AAGCAGCTCTCTAGCACCGTA
siBRCA2-1	GACUCUAGGUCAAGAUUUAAG
siBRCA2-2	GTTTAGAAAGCCAAGCTACTA
